# Colorectal cancer screening in Uruguay: current assessment and roadmap for the future

**DOI:** 10.1186/s41155-021-00178-9

**Published:** 2021-06-29

**Authors:** Micaela Reich, Lydia P. Buki

**Affiliations:** 1 Universidad Católica del Uruguay, Montevideo, Uruguay; 2 University of Miami, Coral Gables, Florida 33146 USA

**Keywords:** Cancer early detection, Cancer screening, Colorectal cancer, Uruguay

## Abstract

Cancer is a leading cause of death worldwide and is expected to remain a public health concern for years to come. Within Latin America, Uruguay has the highest colorectal cancer rates. Heeding past calls to action, in this article we provide a critical assessment of colorectal cancer needs and opportunities in Uruguay with a focus on developing a roadmap for future action. First, we provide an overview of risk factors, screening procedures and guidelines, and screening rates. Next, we provide an overview of psychosocial factors that influence colorectal cancer screening, with the goal of providing guidance for future behavioral health promotion initiatives in Uruguay. In this effort, we present four conceptual models that may be used for interventions: the ecological systems theory, informed decision-making, the health beliefs model, and the health literacy model. Subsequently, we propose using an integrated model based on the ecological systems theory and health literacy model to develop national, local, and community-based interventions to increase screening rates and lower the colorectal cancer burden in Uruguay. We close the paper with a summary and implications section, including recommendations for future research programs focused on the assessment of factors that influence screening.

## Introduction

Cancer is a leading cause of death worldwide (World Health Organization, 2021) and is expected to remain a public health concern for years to come. Population increases and the continued graying of the population, coupled with lifestyle behavior changes associated with greater cancer risk, will contribute to higher incidence and mortality rates (Torre, Siegel, Ward, & Jemal, 2016; World Health Organization, 2021). Colorectal cancer (CRC), in particular, is a leading cause of cancer burden among men and women, ranking third in incidence and second in mortality worldwide, with over 1.9 million new cases and 935,000 deaths annually (World Health Organization, 2021). This cancer does not affect everyone equally; recent trends show that men exhibit higher CRC incidence rates than women (World Health Organization, 2020) and that incidence and mortality rates are increasing among individuals under 50 years of age, resulting in premature death (Siegel et al., 2020).

Higher incidence rates have been observed in Latin America, where they have been historically low (Torre et al., 2016). These increases are attributed to lifestyle changes, including uptake of Westernized diets with lower levels of fiber, sedentary activity patterns, and higher rates of smoking (Torre et al., 2016). In certain areas, low screening rates may contribute to detection at more advanced stages, with lower chances of survival (Abualkhair et al., 2020). Screening rates, in turn, are influenced by national screening policies, recommendations, and health campaigns. Implementing clear and w-considered screening guidelines, offering widespread access to screening, and conducting effective national campaigns would provide an optimal strategy to lower the CRC burden. However, across the 12 countries that comprise South America, in 2016, only Argentina, Chile, Colombia, and Uruguay had established formal screening guidelines (Aedo, Conde, & Pereyra-Elías, 2016).

Within Latin America, Uruguay has the highest CRC rates, with evidence suggesting that more than half of cases (54%) are detected at advanced stages (Garau, Musetti, Alonso, & Barrios, 2019; Ministerio de Salud Pública, 2018). Across all cancers, it is ranked second highest in incidence and mortality among Uruguayan women (28.24 and 12.36 per 100,000, respectively), and third highest among Uruguayan men (38.30 and 19.01 per 100,000, respectively). Strikingly, approximately 4% of Uruguayans will be diagnosed with CRC in their lifetime, with a 5-year survival rate of 55% (Comisión Contra el Cáncer, 2020). This is a very high incidence rate coupled with a relatively low survival rate, despite the fact that CRC is preventable. Not only are there screening tests that can help detect CRC in the early stages, when more treatment options are available, but these tests can also help prevent CRC by detecting polyps at precancerous stages (American Cancer Society, 2020b). With the widespread use of these tools, the high incidence rate can be lowered and survival rates can be increased.

Heeding past calls to action (Organización Panamericana de la Salud Uruguay, 2018), and consistent with national health objectives (Ministerio de Salud Pública, 2017), our goal in writing this article is to contribute toward the amelioration of the CRC burden, which is a major public health issue in Uruguay. We present a critical assessment of the state of the art, promote a call to action, and offer recommendations for increasing screening rates and lowering incidence and mortality rates. In this effort, we provide an overview of CRC risk factors as well as screening procedures, guidelines, and rates. We subsequently review psychosocial factors that are germane to the development of future behavioral health promotion initiatives. To provide guidance on the creation of interventions, we present four conceptual models and their relevance to CRC screening efforts: ecological systems theory, informed decision-making, the health beliefs model, and the health literacy model. Given the limitations inherent in the use of individual models, we propose the use of an integrated model. Based on the ecological systems theory and health literacy model, the integrated model would be culturally relevant and congruent with programmatic needs to lower the CRC burden in Uruguay. We close the paper with a summary and implications section, including recommendations for future intervention and research programs. To begin this analysis, we first provide an overview of the various factors that have fueled the recent increase in cases.

## Risk factors for CRC

The most prominent risk factors for CRC are the standard Western diet (e.g., consumption of red meat and low-fiber foods), a sedentary lifestyle, overweight and obesity, smoking, and alcohol consumption (American Cancer Society, 2020a). Consistent with these risk factors, studies have found that higher CRC rates among men in Uruguay are associated with low intake of plant-based foods (Deneo-Pellegrini et al., 2002) as well as high consumption of red meat, dietary iron, and eggs (De Stéfani et al., 2009). Among women, higher intake of alcoholic beverages and eggs has been associated with a higher risk of CRC (De Stéfani et al., 2009).

In fact, Uruguayans are among the top consumers of beef in the world (World Beef Consumption Per Capita, 2021). In 2019, total meat consumption per person was 87 kg (192 lb), with more than half of that amount corresponding to red meat intake (Instituto Nacional de Carnes, 2019). Meat consumption is an integral part of Uruguayan culture. Most traditional dishes feature red meat as a main ingredient and oftentimes include ham and/or bacon as well. A few of these dishes are *asado* (barbeque), *milanesa* (beef or chicken schnitzel), *chivito* (a popular sandwich made with white bread featuring a variety of ingredients including beef, bacon, eggs, mozzarella cheese, french fries, and generous amounts of mayonnaise), *chorizo al pan* (sausage sandwich on white bread), *empanadas* (filled white flour pastries, which may be fried and typically include meat fillings), *pastel de carne* (a variety of meatloaf with a layer of mashed potatoes), *torta de fiambre* (a ham and cheese tart with egg, and a crust made of white flour), and *sandwich olímpico* (a crustless white bread sandwich that includes ham, cheese, boiled egg, and mayonnaise).

With regard to tobacco, more than 2 in 10 Uruguayans over the age of 15 are consumers (Global Adult Tobacco Survey, 2017). In addition, Uruguay has the second highest rate of whiskey consumption in the world (Spirits International, 2021). Uruguayans have also experienced significant increases in overweight and obesity over the past 10 years, with 65% of adults and 40% of youth exhibiting one of these conditions (Ministerio de Salud Pública, 2019). Finally, consistent with increases in the number of automobiles per capita, over half (55%) of Uruguayans 18 years of age or older live a sedentary lifestyle, with only 37% of individuals over the age of 49 engaging in physical activity on a regular basis (El País, 2016).

Recently, a focus in the literature has been the effects of mate consumption on CRC incidence. Examining mate’s potential carcinogenic effects is especially important in Uruguay, as it is the most traditional drink in the country; few people do not drink mate. Uruguayans consume 10 kg (22 lb) of mate annually per capita (El País, 2020). In previous studies, this infusion was identified as a risk factor for oral, esophageal, and laryngeal cancer (Loria, Barrios, & Zanetti, 2009; Ronco et al., 2004; Sewram, De Stéfani, Brennan, & Boffetta, 2003). In the first epidemiological study of its kind, Ronco et al. (2017) found an inverse relation between mate consumption and CRC incidence rates, suggesting that mate is a protective factor for this cancer type. The aforementioned carcinogenicity found for other cancer types has been linked to the hot temperature at which mate is traditionally consumed rather than to chemical compounds inherent within mate (Ghassan Riachi & Bastos De Maria, 2017). Two additional factors associated with low CRC rates in the literature on Uruguayans are higher vegetable and fruit intake, especially lettuce, apple, and banana consumption (Deneo-Pellegrini, De Stefani, & Ronco, 1996). These findings point to a potential benefit of changing population diets to lower CRC incidence.

Additional CRC risk factors reported in the literature include inflammatory bowel diseases such as Crohn’s disease or ulcerative colitis, a personal or family history of CRC or colorectal polyps, and genetic syndromes such as familial adenomatous polyposis or hereditary nonpolyposis CRC (i.e., Lynch syndrome; American Cancer Society, 2020a; Centers for Disease Control and Prevention, 2021b). In addition, individuals with type 2 diabetes are at higher risk for CRC (American Cancer Society, 2020a). Given the high incidence of CRC in Uruguay, many individuals present a personal or family history of this cancer. Moreover, of 360,000 Uruguayans living with diabetes, 85 to 90% have type 2, the most common form of the condition (*Organización Panamericana de la Salud Uruguay*, n.d.).

CRC symptoms include a change in bowel habits that lasts for more than a few days such as diarrhea, constipation, or narrowing of the stool; a feeling that you need to have a bowel movement that is not relieved by having one; rectal bleeding with bright red blood; blood in the stool, which might make the stool look dark brown or black; cramping or abdominal pain; weakness and fatigue; and unintended weight loss (American Cancer Society, 2020c; American Society of Clinical Oncology, 2019). Given that these symptoms may be identified and followed up with screening tests, and that it is also possible to have precancerous polyps without symptoms, it is imperative to increase awareness and use of screening tests. We discuss available screening procedures next.

## CRC screening procedures

CRC is one type of cancer that can benefit from early detection and, even more importantly, can be prevented through routine screening. This is because it can take 10 to 15 years for any abnormal cells that develop to grow into cancerous polyps. Thus, it is possible to identify and remove premalignant polyps before they become cancerous (American Cancer Society, 2020b). In addition, when polyps are detected early, less invasive and more effective treatment options are available by interrupting the polyp–cancer sequence (American Cancer Society, 2020b; National Cancer Institute, 2020). By enacting population-based CRC screening protocols, incidence, morbidity, and mortality may be effectively reduced (Siegel et al., 2020; World Health Organization, 2021).

Several options for CRC screening are available, although they vary in cost and infrastructure requirements. Stool-based tests check the stool (feces) for signs of cancer. Precancerous lesions or malignant tumors at early stages can be detected because polyps may bleed, and blood can subsequently be found in the stool. These tests check for blood in the stool and have several advantages: they are noninvasive, easy to perform, and relatively inexpensive (American Cancer Society, 2020b). However, they require more frequent screening than other exams. Visual exams, on the other hand, allow health professionals to observe the structure of the colon and rectum for any abnormal areas. The exams are performed either with a scope (a tube-like instrument with a light and very small video camera at the end) that is inserted into the rectum, or with special imaging tests such as X-rays (American Cancer Society, 2020b). One of these exams is a colonoscopy—a highly sensitive test. During the colonoscopy procedure, the doctor examines the colon and rectum in all its length with a colonoscope, which is introduced through the anus into the rectum and colon. In contrast to stool-based exams, these procedures require preparation, are more invasive and more expensive, and require more time, thus creating greater barriers for participation (American Cancer Society, 2020b).

The fecal occult blood test (FOBT), a stool-based test, is used in many parts of the world because it is inexpensive, simple, and noninvasive (Centers for Disease Control and Prevention, 2021a; Torre et al., 2016). For this exam, the person collects a small quantity of feces following the instructions on a collection kit and sends the sample to a laboratory to obtain the results. Should blood be found, it may indicate the presence of a benign or malignant polyp. Thus, a positive result must be followed up with a colonoscopy to identify the cause of the bleeding (American Cancer Society, 2020b). Having reviewed the screening options available, we turn our attention to screening guidelines and rates.

## CRC screening guidelines and screening rates

In this section, we refer to information available for Uruguay and the USA. In Uruguay, national public health authorities recommend FOBT screening every other year for individuals 50 to 74 years old and earlier screening for those with a personal or family history of CRC (Ministerio de Salud Pública, 2018). In fact, 8.5% of cases are detected before the age of 50 in Uruguay (Comisión Contra el Cáncer, 2020).

In the USA, there are two CRC screening guidelines, one promoted by the American Cancer Society (Wolf et al., 2018) and another by the U.S. Preventive Services Task Force (2016). The American Cancer Society recommends that average-risk adults aged 45 years and older undergo regular screening with either (a) a high sensitivity stool-based test every year, (b) a multitargeted stool DNA test every 3 years, or (c) a structural (visual) exam. For the visual exam, they recommend one of three options, based on personal preference and test availability: a colonoscopy every 10 years, CT colonography (a virtual colonoscopy) every 5 years, or flexible sigmoidoscopy every 5 years. As part of the screening process, all positive results on noncolonoscopy screening tests should be followed up with a colonoscopy for diagnostic purposes (Wolf et al., 2018). The U.S. Preventive Services Task Force is currently revising its recommendations to lower the age at which routine screening should occur to 45, following the rise in diagnoses among younger individuals (The New York Times, 2021).

Despite the aforementioned recommendations, low screening rates have been documented among Latinx populations in the USA (Viramontes et al., 2020) and individuals in Uruguay (Ministerio de Salud Pública, 2018). In the USA, Latinx populations have higher CRC mortality rates than non-Latino Whites due to disproportionate rates of detection at more advanced stages (Castañeda et al., 2012, 2020; Efuni, DuHamel, Winkel, Starr, & Jandorf, 2015). In Uruguay, only 42% of individuals ages 50 to 74 have obtained the FOBT. There are gender differences, such that women exhibit higher FOBT screening rates (46%) than men (36%; Poder Legislativo/República Oriental del Uruguay, 2000). The fact that over half of eligible patients is not screening according to recommended guidelines is particularly concerning, as the health system provides free CRC screening exams for all Uruguayan residents (Poder Legislativo/República Oriental del Uruguay, 2000). Because financial access is not a barrier to screening, it is likely that psychosocial determinants may account for the low documented screening rates. We review these next.

## Psychosocial factors that influence CRC screening

Although some data have been gathered on biological and behavioral risk factors for CRC in Uruguayan populations, we did not identify any studies that report on psychosocial factors that act as facilitators or barriers to CRC screening. Instead, given the findings’ likely applicability to Uruguayan populations, we draw from studies conducted with Latinx populations in the USA as well as a couple of international pertinent investigations.

Across various studies, health care providers’ recommendation that the patient obtains a CRC screening exam has emerged as a major facilitative factor (e.g., Byrd, Calderón-Mora, Salaiz, & Shokar, 2019; Gonzalez et al., 2020). Consistent with this finding, trust in physicians has also been identified as a facilitator (Bynum, Davis, Green, & Katz, 2012; Casal, Velázquez, Mejía, Cúneo, & Pérez-Stable, 2009). Health care providers’ recommendation to screen has been associated, in particular, with higher rates of screening among men (Clarke, Sharp, Osborne, & Kearney, 2015).

With regard to obtaining a colonoscopy, in one study of Latinx with moderate CRC risk, those who exhibited greater worry and lower optimism were more likely to obtain the test than individuals with lower worry and higher optimism (Efuni et al., 2018). With regard to stool-based tests, researchers have found that the most common perceived facilitators were live call reminders and postage-paid return envelopes (Ylitalo et al., 2019). Moreover, a social support-based intervention was successful in increasing screening rates. In this study, individuals from the community were invited to bring one or more loved ones to the appointment where they were referred for screening; at the meeting, the loved one signed a pledge to assist the patient in obtaining the screening (Dominic et al., 2020). Also, individuals with higher levels of health literacy have reported more positive attitudes toward screening than those with lower levels of health literacy (Gabel, Larsen, Edwards, Kirkegaard, & Andersen, 2019).

In examining barriers to CRC screening, researchers have found that current smokers are less likely to return a stool-based test than those who do not smoke (Ylitalo et al., 2019). In addition, it is possible that having inaccurate beliefs and knowledge about risk factors for CRC would lead to lower screening rates, if an individual does not feel at risk or does not know about the benefits of screening and early detection. For example, Latinx in the USA inaccurately identified anal sex and strained bowel movements as causal factors for CRC (Goldman, Diaz, & Kim, 2009). More specifically, individuals with low levels of health literacy have exhibited low levels of CRC knowledge (Gabel et al., 2019). Attitudes, beliefs, emotions, and knowledge reported by Latinx populations in the USA include fear, embarrassment, stigma, machismo, fatalism, misperceptions about cancer and CRC risk, unclear instructions from doctors, mistrust of providers, and use of alternative health care as barriers to screening (Bynum et al., 2012; Byrd et al., 2019; Morris, Field, Wagner, Cutrona, & Roblin, 2013; Natale-Pereira et al., 2008; Ritvo et al., 2013; Ylitalo et al., 2019). With regard to stool-based tests in particular, lack of motivation and forgetfulness have been associated with lower levels of screening completion (Ylitalo et al., 2019).

It is critical to take into account the aforementioned factors in the design of interventions that aim to increase screening rates. Unfortunately, despite the importance of having this information as it relates to the Uruguayan population, there is a research gap in this area. Thus, there is a great need to examine psychosocial factors that influence CRC screening uptake in Uruguayan populations in future studies. Despite this lack of information, it is important to begin working toward the implementation of interventions, which would be refined as more data about the Uruguayan population becomes available. In the next sections, we provide a conceptual foundation for the development of programs designed to lower incidence and mortality rates in Uruguay.

## Conceptual models and interventions

The global cancer burden could be substantially reduced by implementing effective interventions in a broad and equitable manner (World Health Organization, 2021). To be successful, interventions must address the population’s needs and values, as well as be guided by theories and models on health behavior (Hilliard, Riekert, Ockene, & Pbert, 2018).

In our review of the literature on interventions designed to increase CRC screening rates in Latinx populations, we noted that many studies attended to cultural issues and/or reported a guiding model for their research, whereas others did not (e.g., Ou et al., 2019). In this section, we aim to provide guidance on those theoretical frameworks that may be used to conceptualize interventions in Uruguay. Although many frameworks have been used in CRC-focused studies (e.g., chronic care model; Castañeda et al., 2020), we selected five for our review based on the following factors: (a) the clear applicability of the framework to CRC screening interventions; (b) the flexibility provided by the framework to attend to cultural values, beliefs, and needs of the Uruguayan population; and (c) the overall feasibility of implementing the framework in Uruguay. The four frameworks are ecological systems theory, informed decision-making, the health beliefs model, and the health literacy model. Next, we briefly introduce each model and illustrate its applicability to CRC screening.

### Ecological systems theory

Bronfenbrenner’s (1979) ecological systems theory, as applied to health promotion, provides a comprehensive way of conceptualizing the multiple areas that influence behavioral outcomes. These areas include individual factors such as beliefs, microsystem-level interpersonal, and community factors such as family support, mesosystem-level interactions across microsystem factors such as health care provider and work schedules, exosystem-level organizational factors such as health care systems, and macrosystem-level larger cultural ideologies and social norms such as *machismo*. Interactive effects across areas lead to the identification of leverage points to enhance health-promoting behaviors. By designing combined interventions that focus at all levels of the model, the effectiveness of public health programs may be optimized (Unicef, n.d.).

Based on ecological systems theory, Arredondo et al. (2020) implemented a health promoter-led intervention to address individual, interpersonal, community, and organizational levels among a community sample of individuals overdue for CRC screening. Participants were Latinx men and women in the USA between the ages of 50 and 75. The intervention, which was carried out across various community-based organizations, included the delivery of a 2.5-h workshop followed by assistance from a health promoter at 2 weeks, 6 months, and 9 months after the workshop, to help in making the screening appointment if needed. Screenings were provided at a federally qualified health center.

At the intrapersonal level, the intervention sought to increase participants’ knowledge of CRC prevention as well as to address attitudes and barriers related to screening. At the interpersonal level, participants were provided social support during the workshop and at 2 follow-up points, to assist those who needed help in scheduling the screening. Focusing on the community level, collaborations with various community entities such as schools, senior centers, and recreational centers allowed the workshops to be delivered in settings familiar to the participants, with health promoters providing a “warm handoff” to the clinic. Finally, at the organizational level, the delivery of workshops and outreach by health promoters created and/or strengthened the link between the participants and the medical clinic.

The intervention, titled *Juntos Contra el Cáncer*/Together Against Cancer, consisted of a single group, pretest–posttest design with no random assignment or control. Results were in the expected direction, although they need to be interpreted within the context of limitations in the research design. The participants reported statistically significant increases in knowledge about CRC screening and statistically significant decreases in perceived barriers to screening after the intervention. Of the 177 participants who were overdue for screenings, 118 (67%) reported completing the screening within the 9 months following the intervention.

### Informed decision-making

The goal of informed decision-making is for the patient to understand the target disease or condition as well as treatment options including their benefits, risks, limitations, alternatives, and uncertainties (Sheridan, Harris, & Woolf, 2004). In addition, in making a decision, individuals would have considered their own preferences (when options are available) and would have perceived that they participated in decision- making at a level that is comfortable to them. More specifically, when making a decision about cancer screening, individuals actively need to (a) understand the screening test, its risks, benefits, and alternatives; (b) understand their personal values and preferences; (c) weigh the benefits and risks of obtaining the screening; (d) understand their preferences for collaborative or individual decision-making; (e) access additional information when needed; and (f) decide on an action plan (Sheridan et al., 2004). In a meta-analysis of studies using decision aids to promote CRC screening, scholars found that patients exposed to a decision aid showed greater knowledge than those exposed to a control condition. However, contrary to expectations, no differences in screening interest or behavior were found across conditions (Volk et al., 2016). Therefore, to illustrate this framework, we discuss a study that included not only a focus on decision-making but also on social support; this combination of strategies showed a significant increase in screening rates.

In a study of 400 Latinx patients overdue for CRC screening (Myers et al., 2019), participants were randomly assigned to one of two treatment conditions: (a) standard treatment or (b) decision support and navigation intervention. To gain insight into the application of the framework, we will discuss the study procedures in more detail. Initially, participants in both conditions underwent several similar processes. First, they were given descriptions of stool-based and colonoscopy screening options, to ensure they understood the screening tests. Subsequently, researchers asked all participants about their decision to screen (i.e., screen, not screen, considering screening, undecided). Participants in both conditions also received a standard set of materials: a letter from their primary care doctor encouraging screening, instructions for arranging either test, and informational print materials in English and Spanish about the tests’ procedures, risks, and benefits. Participants in the decision support condition subsequently received a call from a patient navigator to discuss barriers to screening and to help them weigh the benefits and risks of obtaining the screening based on their personal values and preferences. Next, using computer software, patient navigators worked with participants in the intervention condition on the design of an action plan for screening that addressed these barriers. Twelve months postintervention, 43% of participants in the standard condition received a screening compared to 78% in the decision support arm.

### Health belief model

As the name suggests, in this model beliefs are posited to be central drivers of health promotion behavior (Rosenstock, 1974). According to this model, individuals’ decision to screen for CRC would be influenced by their beliefs related to (a) their susceptibility to developing CRC, (b) CRC’s severity or seriousness, (c) benefits from CRC screening, and (d) barriers to CRC screening (Janevic & Connell, 2018). In addition, behavior is expected to be influenced by triggers that stimulate the behavior, called cues to action. In a systematic review of articles that applied the model to CRC screening specifically, researchers found significant support for the contribution of the main variables to screening intention or behavior (Lau, Lim, Wong, & Tan, 2020). In addition, they found that other cultural, intrapersonal, interpersonal, and policy variables were associated with screening outcomes in some studies and recommended that these additional factors be considered when designing interventions to increase CRC screening uptake.

To illustrate the use of this model, we draw on a larger study conducted by Mojica, Almatkyzy, and Morales-Campos (2021) related to preventable cancers. In this study, the authors used a pretest–posttest design with a control group with 442 Latino participants ages 50 to 75 who had never obtained a CRC screening test. Specifically, the study was designed to test the effectiveness of a psychoeducational program plus navigation. Participants in the control and treatment conditions took part in the educational presentation, which included information about individuals’ susceptibility to developing CRC, CRC’s severity and seriousness, and benefits of CRC screening. Individuals in the treatment condition also received navigation assistance from community health workers to address barriers to screening, such as filling out paperwork, scheduling medical appointments, and providing reminders (a cue to action). Tests were provided for free; therefore, cost was not a barrier to screening for participants in this study.

Results showed that for individuals in the treatment condition, knowledge about screening guidelines increased; in addition, these participants reported changes in (a) beliefs about benefits of early screening and (b) self-efficacy related to changing cancer outcomes. Overall, men and women in the intervention group obtained CRC screenings at higher rates than participants in the control group.

### Health literacy model

Health literacy refers to “the degree to which individuals have the capacity to obtain, process, and understand basic health information and services needed to make appropriate health decisions” (Ratzan & Parker, 2000). As applied to CRC screening, the model posits that an individual’s print literacy, oral literacy, numeracy skills, and cultural and conceptual knowledge related to CRC and CRC screening would influence lifestyle choices and screening outcomes. Cultural and conceptual knowledge would refer to the beliefs, attitudes, emotions, and knowledge a patient holds about CRC (Buki, Yee, Weiterschan, & Lehardy, 2016). The model suggests three points of intervention: culture and society, the health system, and the educational system (Institute of Medicine, 2002). Given the relatively recent development and refinement of this model (Nutbeam, McGill, & Premkumar, 2018; Walters, Leslie, Polson, Cusack, & Gorely, 2020), we were unable to identify an example of a health literacy-based CRC intervention focused on Latinx populations. However, the model shows promise, as adequate health literacy has been associated with greater odds of CRC screening in British samples (Kobayashi, Wardle, & von Wagner, 2014). Moreover, in terms of numeracy, adults with the highest comprehension scores when reading a basic medicine label were found to have 20% greater odds of participating in screening than those who responded incorrectly to at least one item (Kobayashi et al., 2014). Overall, in a review of 22 studies that used health literacy as the basis for an intervention to enhance various health outcomes, participants reported higher levels of health literacy in 15 studies (77%), and improved behavioral health outcomes in 7 of 8 studies (88%) that measured them (Walters et al., 2020). The results are all the more encouraging for use with a Uruguayan population because the 22 studies reviewed were conducted across nine countries, and participants included adults and migrant populations, suggesting the applicability of the model cross-culturally.

## Summary, implications, and recommendations for CRC interventions in Uruguay

In summary, CRC is a leading cause of cancer burden worldwide. In addition, its incidence is expected to increase in the next 25 years, especially in less developed countries, in part due to the adoption of Western lifestyle patterns. In this context, primary prevention, which focuses on interventions to change health-related behaviors, will be key to address the anticipated rise in CRC burden (Rabeneck, Chiu, & Senore, 2020).

In particular, CRC is a major public health concern as a leading cause of cancer morbidity in Uruguay. Thus, the Uruguayan government has designated the lowering of CRC morbidity and mortality as a national health objective (Ministerio de Salud Pública, 2017). Given its high prevalence, we speculate that virtually every person in Uruguay knows someone who has been diagnosed with the condition or will be diagnosed themselves. As we documented in this review, Uruguayans exhibit several important modifiable factors that heighten their risk for CRC including a Western diet, a sedentary lifestyle, alcohol consumption, tobacco use, overweight, obesity, and type 2 diabetes. Thus, it is imperative for researchers and public health professionals to prioritize and focus resources to effectively change those factors which may respond to programmatic efforts.

**Fig. 1:**
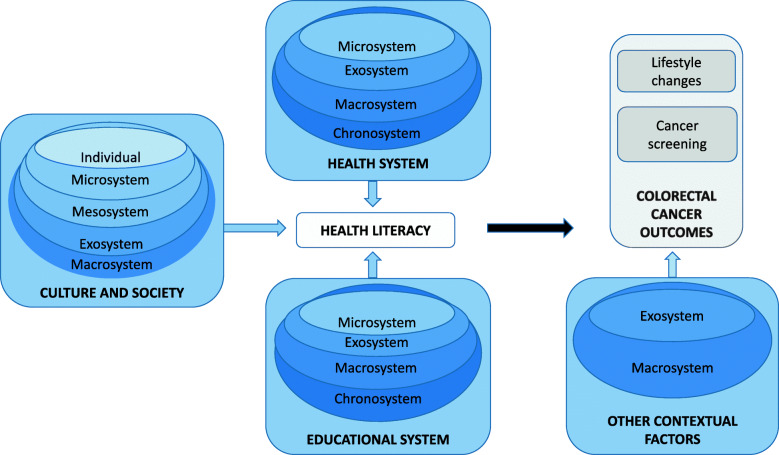
Integration of ecological theory and the health literacy model depicting factors that influence CRC outcomes

We recommend approaching the task of lowering incidence and mortality rates by having a two-pronged approach. First, interventions are needed to lower incidence rates by changing lifestyle factors such as diet and exercise. Second, interventions are needed to increase screening rates. Because there are several tests available that may help prevent or detect CRC in the early stages, investing in a screening infrastructure, including screening promotion, is critical. These two approaches should be implemented at the national, local, and community levels. The most comprehensive conceptual framework for the programs would be an integration of ecological systems theory and the health literacy model (see Fig. 1). This framework allows for the examination of risk factors and psychosocial determinants of health behaviors at various ecological levels, as well as the identification of potential causal pathways that influence outcomes.

As shown in the figure, to be optimally effective, interventions should promote change at various ecological levels. In addition, based on the health literacy model, we propose that researchers prioritize three points of intervention: (a) culture and society, (b) the health system, and (c) the educational system. Specifically, interventions focused on culture and society would need to address the individual (e.g., attitudes and beliefs), microsystem (e.g., doctor’s screening recommendation), mesosystem (e.g., a doctor’s screening recommendation to a family member), exosystem (e.g., steps required to complete FOBT testing), and macrosystem (e.g., cultural attitudes toward prevention) levels. In turn, interventions focused on the health and educational systems would address the microsystem (e.g., sending a reminder to patients overdue for screenings), exosystem (e.g., physicians’ exposure to preventive models in medical training), macrosystem (e.g., transmission of cultural traditions related to eating red meat), and chronosystem (e.g., national screening guidelines) levels. In addition, we posit that other contextual factors influence CRC outcomes at the exosystem (e.g., exposure to preventive messages through mass media) and macrosystem (e.g., infrastructure for availability of fresh fruit) levels, which must be addressed through intervention.

There are several factors that render this a favorable time to develop national screening initiatives in Uruguay: in 2016, a CRC public health campaign was launched in Montevideo, called *Campaña 2016 del Plan Nacional de Prevención y Detección Precoz de Cáncer Colo-Rectal* (S. Sanabia, personal communication, July 23, 2020). This campaign consisted of scripted guided tours of an inflatable giant colon located at the front of the city’s town hall for a couple of months, with the objective of increasing CRC knowledge and screening intention. Although no formal evaluation of the campaign was conducted, it reflects the government’s priority to lower CRC incidence and mortality, and was sufficiently prominent that it provides a foundation for additional efforts. Another positive factor in the development of screening campaigns is that Uruguay has a public health system that ultimately provides health insurance coverage to those who do not have it through other sources. Thus, the cost of CRC exams is not a barrier for screening. Without this barrier, and continuing with the recent impetus to educate the public, Uruguay provides a fruitful context to focus on modifiable factors such as psychosocial and sociocultural determinants of CRC and CRC screening.

In preparation for interventions, whether national, local, or community-based, the lack of data to inform programming needs to be addressed. We call for research efforts to conduct a systematic assessment of the population in order to explore and understand individuals' knowledge, beliefs, attitudes, barriers, facilitative factors, and screening behaviors related to CRC. In addition, this comprehensive assessment should gather data about contextual factors that may contribute to screening disparities such as age, formal education level, or geographical region. Based on the systematic assessment of CRC and identified educational needs, psychoeducational programs and allied interventions could be tailored to specific Uruguayan subpopulations.

One major obstacle to surmount in conducting the aforementioned assessment is the lack of measures designed to assess these psychosocial factors in a valid and reliable manner. Thus, we recommend the development of a measure to assess CRC health literacy tailored specifically for the population. Such a measure, which would be available in Spanish, should be based on focus group data about the population’s cultural attitudes and beliefs related to CRC and CRC screening. It should also reflect patient interactions with the health care system relevant to the Uruguayan population. The measure should assess CRC-related knowledge and should be tailored to reflect the CRC screening tests and guidelines promoted by the Uruguayan health system. For example, currently individuals who obtain the FOBT are asked to obtain a stool specimen and place it in the refrigerator until they take it to the lab for analysis. This may serve as a barrier for people who are uncomfortable with this required process. The measure should also be reviewed by local health care providers and community experts to ensure its relevance and accuracy within the Uruguayan context. The measure would have undergone large-scale psychometric testing with individuals 50 to 74 years of age and would assess various factors that influence screening rates (e.g., patients’ beliefs about doctors’ effectiveness at curing disease) as well as the population’s knowledge, beliefs, attitudes, emotions, barriers, facilitative factors, and screening behaviors related to CRC. For example, knowledge items may assess knowledge about signs and symptoms, risk factors, protective factors, and prognosis. Other items may address beliefs about benefits of early screening, perceived susceptibility to CRC, benefits from CRC screening, barriers to CRC screening, and cues to action. Finally, we recommend assessing individuals’ self-efficacy related to changing a cancer outcome.

With this information, programs can be designed for optimal impact. The most comprehensive conceptual framework for the programs, as mentioned previously, would be an integration of the ecological systems theory and health literacy model. This would be consistent with previous recommendations that interventions be designed across various ecological levels. In addition, using the health literacy model would allow not only for an examination of the main constructs in various health promotion frameworks and theories (e.g., knowledge of screening tests for informed decision-making; perceived susceptibility in the health behavior model), but also would provide guidance for interventions to influence culture and society, the health system, and the educational system. We would expect this broad contextual approach to intervention to be effective in Uruguay for a number of reasons. First, although data about Uruguayans’ knowledge, beliefs, attitudes, and emotions are not available, it is likely that some of the aforementioned findings will replicate with this population. Having a health care provider recommendation is the single most important correlate of screening, and to ensure that patients obtain the recommendation, interventions will be needed within the culture and society context (e.g., at the interpersonal ecological level), as well as at the health system and educational levels. In addition, cues to action such as reminders to do the test, which have been shown to increase screening rates, would be consistent with designing an intervention within the health systems context (e.g., at the exosystem ecological level). Further, knowledge, beliefs, attitudes, and emotions would be addressed at the culture and society intervention point. The content of the intervention (e.g., a focus on knowledge about the importance of CRC screening) would be based on the data obtained from the national assessment.

With data from the assessment, educational materials and dissemination approaches can be optimally tailored. National, local, and community campaigns would be evaluated and modified as needed, to ensure their effectiveness not only at promoting healthier lifestyle choices and increasing screenings among those who have never screened, but also at increasing rescreening rates among those who are overdue. We hope that through this critical review, researchers, practitioners, and policy makers will be prompted to heed our call to action. Initiatives and funds need to be prioritized to address this pressing health issue.

## Conclusion

The amelioration of the CRC burden in Uruguay is complex and requires attention to CRC risk factors (e.g., high intake of red meat, sedentary lifestyle) as well as screening procedures, guidelines, and rates. It also requires attention to complex psychosocial factors that would require significant efforts to change (e.g., health care professionals’ disposition; patients’ health literacy, mistrust of the health system, and lack of motivation). A comprehensive, systematic assessment of the population is needed to inform future efforts. Although more research and guidance are needed, there is sufficient evidence to engage in public health action today. We propose the use of an integrated model based on ecological systems theory and the health literacy model; this integrated framework would allow researchers to consider all relevant ecological levels while facilitating behavior change. Using this integrated model, national, local, and community-based interventions may be developed to (a) lower incidence rates by changing lifestyle factors such as diet and exercise, and (b) increase screening and rescreening rates. More action is needed. In this effort, the aforementioned recommendations may serve as a foundation for the amelioration of the CRC burden in Uruguayan populations. We hope this call to action and roadmap will promote research and help shape future efforts that contribute toward lowering CRC incidence and mortality rates in Uruguay.

## Data Availability

Data sharing is not applicable—no new data generated.

## Abbreviations

CRC: Colorectal cancer
FOBT: Fecal occult blood test
